# Novel *Rickettsia rickettsii* Proteins with Potential Use for Serological Detection of Wild Natural Reservoirs

**DOI:** 10.3390/pathogens15090994

**Published:** 2026-09-20

**Authors:** Hernan H. M. da Costa, Maribel L. Loza, Marcia G. Guereschi, Gustavo C. Amorim, Andreza C. Matias, Marcelo Lancellotti, Ana Carolina P. Sousa, Carolina F. Osava, Leonardo J. Richtzenhain, Marcelo B. Labruna, Matias Pablo J. Szabó, Carlos R. Prudencio, Jair P. Cunha-Junior

**Affiliations:** 1 Multi-Purpose Laboratory, Butantan Institute, Sao Paulo 05503-900, Brazil; hhermes@usp.br (H.H.M.d.C.); carlos.prudencio@butantan.gov.br (C.R.P.); 2 Graduate Program Interunits in Biotechnology, University of São Paulo, Sao Paulo 05508-090, Brazil; gucamm@usp.br; 3 Immunology Center, Adolfo Lutz Institute, Sao Paulo 01246-000, Brazil; mgueres@yahoo.com.br; 4 Department of Immunology, Federal University of Uberlandia, Uberlandia 38405-302, Brazil; limachilozamaribel@gmail.com; 5 Center of Mathematics, Computation and Cognition, Federal University of ABC, Santo Andre 09280-560, Brazil; andreza.matias@ufabc.edu.br; 6 Faculty of Pharmaceutical Sciences, State University of Campinas, Campinas 13083-871, Brazil; marcelo.lancellotti@fcf.unicamp.br; 7 School of Veterinary and Animal Science, Federal University of Uberlandia, Uberlandia 38410-337, Brazil; carolsousa.enf.ufu@hotmail.com (A.C.P.S.); carolinaosava@iftm.edu.br (C.F.O.); szabo@famev.ufu.br (M.P.J.S.); 8 Department of Preventive Veterinary Medicine and Animal Health, School of Veterinary Medicine and Animal Science, University of São Paulo, Sao Paulo 05508-270, Brazil; leonardo@usp.br (L.J.R.); labruna@usp.br (M.B.L.)

**Keywords:** *Rickettsia rickettsii*, recombinant proteins, serological detection, wild natural reservoirs

## Abstract

The identification of areas where pathogenic *Rickettsia* spp. circulate, using rapid and low-cost serological approaches in sentinel species, may provide essential information for public actions to prevent, diagnose and appropriately treat rickettsiosis. Therefore, in this study we aimed to produce and characterize novel recombinant proteins from *Rickettsia rickettsii* with potential use in serological detection. The proteins were expressed in *Escherichia coli*, used in mouse immunization protocols, and also evaluated by indirect IgG ELISA using a cohort of serum samples from wild boars naturally exposed to *Rickettsia* spp. and from tick-naive farm pigs. The proteins displayed high immunogenicity with the induction of specific murine IgG antibodies, with high avidity after the third dose of immunization. A high IgG seropositivity rate (100%) and antibody avidity (>70%) were observed in wild boar serum samples when evaluated by ELISA. In addition, a significant positive correlation was observed between the swine-specific IgG levels against rPhenAl and rYJBI and the IgG levels against the rOmpA antigen. The novel proteins produced represent promising new immunogenic and antigenic candidates for serological detection of wild animals naturally exposed to *Rickettsia* spp.

## 1. Introduction

Brazilian Spotted Fever (BSF) is a tick-borne disease that has been recognized in Brazil since 1929; like Rocky Mountain Spotted Fever (RMSF), BSF is caused by *Rickettsia rickettsii* and presents clinical manifestations similar to those of RMSF [1,2]. BSF is an important zoonosis with high case-fatality rates in humans (≥50% of cases) [3,4].

Clinical manifestations are the basis for suspecting BSF, but the symptoms can make diagnosis difficult, especially in the initial phase, as they are notoriously non-specific and can mimic benign viral diseases. Initial symptoms include high fever, rash, headache, severe myalgia, general malaise, nausea and vomiting [5]. The clinical manifestations are variable and difficult to correlate with the geographical and epidemiological context and the presence of the vector, making the clinical diagnosis uncertain. In addition, there is evidence that some Rickettsia infections may present atypical clinical features and reduced or no pathogenicity. Serological cross-reactions may occur, especially between *R. rickettsii* and *R. parkeri*, making accurate identification of the infectious etiologic agent difficult [6]. As BSF is a multisystemic disease, it can present a variable clinical course, ranging from classic cases to atypical forms without rash, and due to the lack of rapid diagnosis, some cases may evolve to fatal outcomes.

Rickettsia bacteria are responsible for diseases in animals and humans worldwide. However, there is a significant gap in knowledge about their circulation and ecology among wild animals and human populations at increased risk of transmission in Brazil. Brazilian wild boars, or feral pigs, constitute an invasive exotic species derived from the European wild boar (Sus scrofa), in all its forms (native, domestic, wild and crossbred), lineages, breeds and different degrees of crossbreeding with domestic pigs. Previous reports demonstrated that the seropositivity for *Rickettsia* spp. was higher in wild boars than in humans and dogs [7]. Although humans are generally regarded as being less exposed to ticks (and therefore *Rickettsia* spp.) than animals, anthropogenic activities such as hunting may increase the risk of exposure and, consequently, the development of tick-borne diseases [8].

The laboratory diagnosis of rickettsioses is based primarily on molecular and serological methods. Serological tests are generally simple and cost-effective and are widely used for confirming rickettsial infections, for conducting seroepidemiological studies and also for differentiating rickettsial isolates. The main disadvantage of serology approaches is their inability to provide detection of Rickettsiae at the species and clade levels. Therefore, the identification and characterization of novel *R. rickettsii* antigens, which may also function as new targets for the serological detection of naturally exposed wild animals, may be of great importance for planning programs to prevent neglected vector-borne diseases [9,10,11].

Our group previously identified several *R. rickettsii* immunoreactive proteins using phage-display technology [12], including the 190 kDa outer membrane protein A (OmpA), phenylalanyl tRNA ligase subunit beta (PhenAl) and a pentapeptide repeat-containing protein, described as “uncharacterized protein YjbI” (YJBI). Since the molecules PhenAl and YJBI had not previously been produced, in this study we characterized these novel *Rickettsia rickettsii* proteins for their potential application in the serological detection of wild natural reservoirs, which may provide essential information for public health actions to prevent, diagnose and appropriately treat this infectious disease.

## 2. Materials and Methods

### 2.1. Bacterial Cells, Plasmids and Serum Samples

The *Escherichia coli* BL21 (DE3) pLysS strain was cultured in Luria–Bertani (LB) medium and used in this study. The pET32a plasmids carrying the *R. rickettsii* genes phent (full length; accession number: WP_014365863.1) and YjbI (full length; accession number WP_012151017.1), as well as a genetic construct encoding a small version of outer membrane protein A (OmpA fragment derived from Swiss-Prot: P15921.1), were designed by Dr. Carlos R. Prudencio [12]. The gene encoding the OmpA fragment (OmpA f), harboring seven immunodominant sequences, was previously characterized by our group [12]. The rationale for selecting the recombinant proteins used in this study is highlighted in Supplementary Figure S1. Serum samples from 31 wild boars naturally exposed to tick infestation and previously identified as seroreactive to spotted fever group rickettsiae by in-house indirect immunofluorescence (using whole-cell *R. rickettsii* and *R. parkeri* antigens [8,13]) were included in this study (Supplementary Figure S2). In contrast, eight serum samples from tick-naive farm pigs intensively raised in enclosed stalls under strict sanitary conditions were used as negative controls. All serum samples were obtained from the Laboratory of Ixodology at the Federal University of Uberlandia, Minas Gerais, Brazil. Mouse serum samples were obtained after mouse immunization in the animal facility of the Interdisciplinary Procedures Center at the Adolfo Lutz Institute.

### 2.2. Animals and Ethics Statements

Female BALB/c mice (6–8 weeks old) were obtained from the animal room of the Interdisciplinary Procedures Center at the Adolfo Lutz Institute. All mice were housed in a mini-isolator with controlled temperature and light cycles, using a Ventilife Ventilated Mouse Rack (Alesco, Monte Mor, SP, Brazil), at 25 °C. The mice were maintained with antibiotic-free food and water *ad libitum* in the experimental animal room of the Immunology Center at the Adolfo Lutz Institute. All animal procedures were previously approved by the Committee for Animal Care and Use (CEUA/UFU 097/13, approved on 27 May 2013, and CEUA/IAL 03/23, approved on 18 July 2023) in accordance with the ethical principles and relevant guidelines for animal research and the ARRIVE 2.0 guidelines [14].

### 2.3. In Silico Analysis of rOmpA, rPhenAl and rYJBI Proteins

Three immunoreactive proteins were previously detected through a phage-display approach at the Adolfo Lutz Institute and the University of São Paulo [12]. The structural prediction of the proteins rOmpA f, rPhenAl and rYJBI was based on the AlphaFold2 deep-learning model using their sequences [15]. The structure images were generated using UCSF ChimeraX v1.6 [16]. The proposed models incorporate energy relaxation, which mitigates three-dimensional imperfections, yielding energetically favorable and physically plausible structures. As a corroborative measure, each protein was analyzed using the ProtParam ExPASy [17] and VaxiJen v2.0 servers [18]. We also compared *R. rickettsii* protein sequences (rOmpA f, rPhenAl and rYJBI) with the corresponding sequences from other *Rickettsia* species using the Basic Local Alignment Search Tool for Proteins (BLASTP) [19] (Supplementary Figure S3).

### 2.4. Expression and Purification of Recombinant Proteins rOmpA f, rPhenAl and rYJBI

The recombinant proteins (rOmpA f, rPhenAl and rYJBI) were expressed in *E. coli* transformed with pET-32a plasmids following an adapted protocol [20]. Protein expression was induced by the addition of 1 mM isopropyl-β-D-1-thiogalactopyranoside (IPTG, Invitrogen™, Carlsbad, CA, USA) at 37 °C for 4 h. Bacterial pellets were collected by centrifugation and lysed by sonication in the presence of lysozyme and Benzonase^®^ nuclease (Sigma-Aldrich, St. Louis, MO, USA) and then processed with sequential treatments of N-Lauryl sarcosine sodium salt (Sigma-Aldrich) and Triton X-100 (Sigma-Aldrich), following an adapted protocol [21,22]. Protein purification was performed using a 5 mL HisTrap™ High-Performance Column (GE Healthcare Life Sciences, Chicago, IL, USA) on a Fast Protein Liquid Chromatography system (ÄKTA™ Pure, Cytiva Life Sciences, Marlborough, MA, USA). Elution was achieved using a linear imidazole gradient (0–500 mM). The purified proteins were dialyzed, concentrated and quantified using the BCA assay. The proteins were analyzed by SDS-PAGE, and ImageJ v1.53 software was used to evaluate the densitometric profiles of the purified recombinant proteins [23,24].

### 2.5. Immunization of BALB/c Mice

For immunization, four groups of four mice were used, and each group received three antigen doses subcutaneously by dorsal injection at 21-day intervals. For each dose, 10 µg of recombinant protein (rOmpA f, rPhenAl and rYJBI) diluted in sterile PBS containing 50% (*v*/*v*) complete Freund’s adjuvant (for the first dose) or incomplete Freund’s adjuvant (for the second and third doses) was used. The mock group received only sterile PBS with the appropriate Freund’s adjuvant. Blood was collected via the submandibular vein on days 0 (pre-immune), 21, 42 and 62. Serum samples were obtained by centrifugation (3000× *g* for 15 min at room temperature) and stored at −20 °C until use. All mice were euthanized three weeks after the third dose under deep anesthesia using ketamine/xylazine (140 mg/kg and 10 mg/kg, respectively).

### 2.6. Indirect IgG ELISA and Avidity Measurement

Briefly, high-binding 96-well plates (Nunc MaxiSorp™ flat-bottom, Thermo Fisher Scientific™, Waltham, MA, USA) were coated overnight at 4 °C with 1 μg/well of recombinant protein diluted in carbonate buffer (60 mM, pH 9.6). On the next day, the plates were washed three times with PBST (PBS supplemented with 0.5% Tween 20 [*v*/*v*]) and then incubated with 100 μL/well of blocking buffer (PBST containing 5% skim milk powder [*m*/*v*]) for 1 h at room temperature. All washing steps were performed using an ELISA plate washer (Washwell plate, Robonik, Thane, India). The plates were then washed three times with PBST and incubated for 1 h at room temperature with 50 µL of serum samples from immunized mice, wild boars or pigs, diluted 1:100 in PBST supplemented with 1% skim milk powder (*w*/*v*). Next, the plates were washed three times with PBST; half of each plate was incubated with 1.5 M potassium thiocyanate (KSCN) and the other half was incubated with PBST (50 µL/well) for 20 min at room temperature. Afterwards, the microplates were washed three times with PBST and incubated with peroxidase-conjugated goat anti-pig IgG (1:1000; Invitrogen™) or anti-mouse IgG (1:5000; Santa Cruz Biotechnology™, Dallas, TX, USA) for 1 h at room temperature. The plates were washed three times with PBST and incubated for 15 min at room temperature with 50 µL/well of One-Step Solution-Linear TMB (Scienco Biotech, Lages, SC, Brazil). The reaction was stopped by adding 50 µL per well of 1 N sulfuric acid (Sigma-Aldrich). Absorbance at 450 nm, with 630 nm as the reference wavelength, was measured using an MB-580 ELISA reader (Heales^®^, Shenzhen, GD, China). The absorbance values obtained from mouse pre-immune serum or farm pig serum samples were used to calculate the cut-off as the mean plus three standard deviations. The ELISA results were expressed as the ELISA index (EI; absorbance/cut off). The avidity index (AI) was calculated as follows: the absorbance value of the KSCN-treated sample was divided by that of the corresponding untreated samples and multiplied by 100%, as previously described [25].

### 2.7. Statistical Analysis

Data were analyzed using GraphPad Prism 10.6 software (GraphPad Software, San Diego, CA, USA). All values were evaluated for normal distribution using the Shapiro–Wilk normality test. The ELISA values were expressed as ELISA index (EI), and the avidity indexes (AI) were expressed as percentage. The AI values from mice were reported as the mean with standard deviation (SD) or the median with range, when appropriate. Statistical significance among the EI values was determined by two-way ANOVA with the Geisser–Greenhouse correction and Tukey’s multiple-comparison test, and significance for AI was determined by paired one-way ANOVA with the Geisser–Greenhouse correction and Tukey multiple-comparison test or the Friedman test with Dunn’s multiple-comparison test, when appropriate. Differences in antigen-specific IgG levels between boars and farm pigs were assessed using the unpaired *t* test with Welch’s correction or unpaired Mann–Whitney test, when appropriate. The avidity indexes (AI) from boars and farm pigs were also expressed as percentage and analyzed by paired one-way ANOVA with the Geisser–Greenhouse correction and Tukey’s multiple-comparison test. Correlations among swine seroreactivity to the rOmpA f, rPhenAl and rYJBI proteins were assessed using Pearson’s correlation test. *p*-values < 0.05 were considered statistically significant.

## 3. Results

### 3.1. In Silico Analyses of Biochemical Properties of Recombinant Proteins from Rickettsia rickettsii

**Figure 1:**
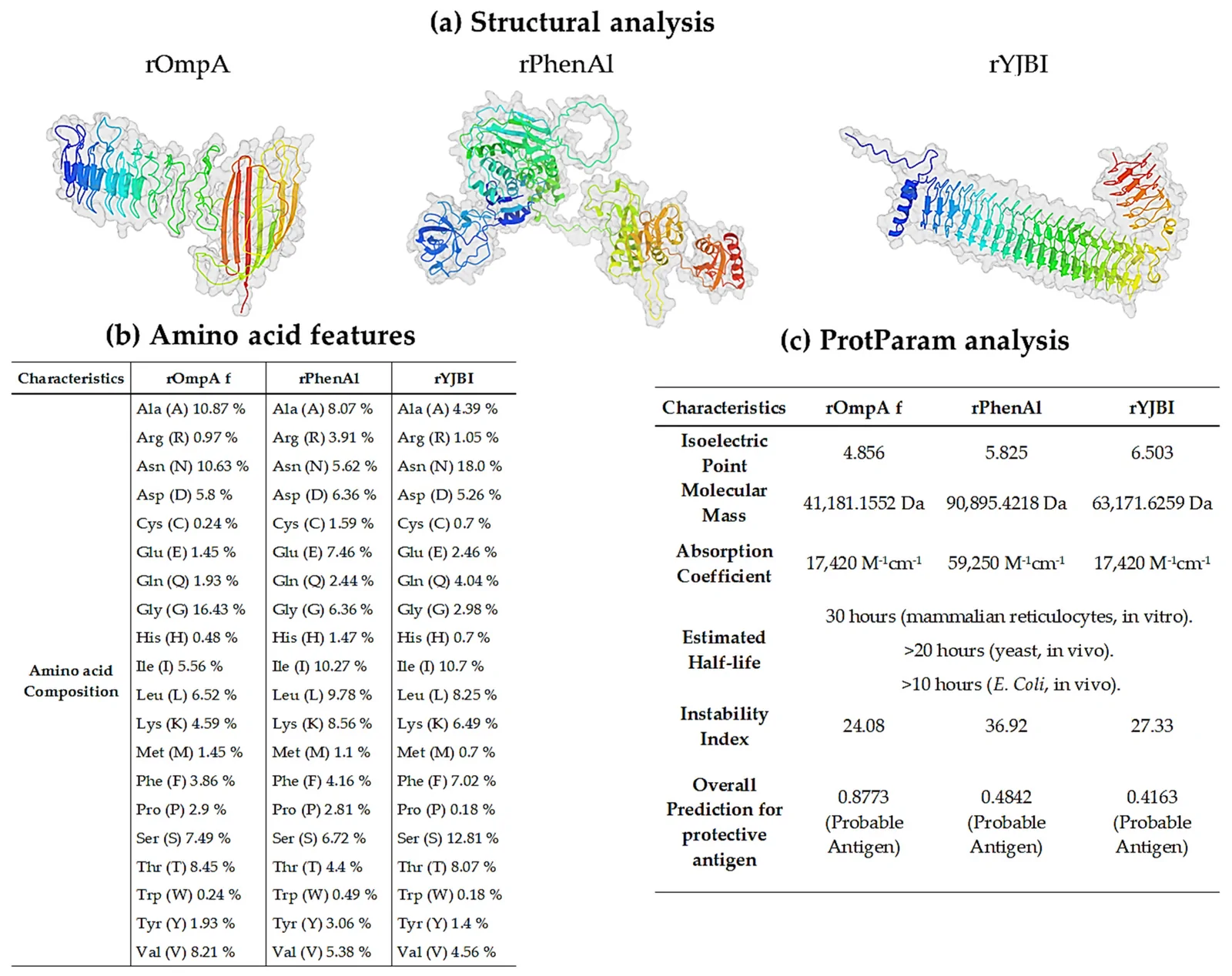
Bioinformatic analysis of the biochemical properties of the rOmpA fragment, rPhenAl and rYJBI proteins. (**a**) Protein modeling using a ribbon diagram representation. The transition from blue to red reflects the path of the polypeptide chain from the N-terminus to the C-terminus, respectively. (**b**) Amino acid composition of recombinant proteins. (**c**) In silico analysis of the biochemical properties of recombinant rOmpA f (immunodominant fragment), rPhenAl and rYJBI.

The OmpA protein is a large protein (190 kDa) composed of multiple repetitive sequences; therefore, in our work we designed a small OmpA protein (fragment) comprising seven immunodominant sequences (herein denominated as rOmpA f) [12]. The prediction of the rOmpA f structure revealed short antiparallel β-sheets in the N-terminal domain and antiparallel β sheets in the C-terminal domain, arranged in a β-barrel conformation. The N-terminal and C-terminal domains are interconnected by loops that confer potential structural flexibility (Figure 1a). The rPhenAl structure revealed multiple α-helices and β-sheets organized in three globular domains, interconnected by flexible loops that confer mobility. This structure may be involved in biosynthetic pathways, allowing conformational adjustments for interaction with potential substrates and cofactors (Figure 1a). Otherwise, the rYJBI molecule displays an elongated and modular structure, characteristic of the Pentapeptide Repeat Protein (PRP) superfamily, with pentapeptide repeats forming a helical “hockey stick” structure stabilized by parallel β-sheets. The rYJBI also displays a small unfolded sequence and short α-helix (amino acids 19–35) at the N-terminal region (Figure 1a).

ProtParam analysis of the rOmpA, rPhenAl and rYJBI proteins revealed differences in their physicochemical properties (Figure 1b,c). The isoelectric points ranged from 4.856 (rOmpA) to 6.503 (rYJBI), indicating acidic to slightly acidic profiles. The computationally calculated molecular masses were 41.18 kDa (rOmpA), 90.89 kDa (rPhenAl) and 63.17 kDa (rYJBI). The absorption coefficient was higher for rPhenAl (59.250 M^−1^cm^−1^) than for the other molecules (17.420 M^−1^cm^−1^). The estimated half-lives were similar among them: 30 h in mammalian reticulocytes (in vitro), >20 h in yeast (in vivo) and >10 h in *E. coli* (in vivo). The instability indices indicated that rOmpA f (24.08) and rYJBI (27.33) are stable proteins, while rPhenAl (36.92) has moderate stability. Prediction as protective antigens indicated that rOmpA f was the most promising (0.8773), followed by rPhenAl (0.4842) and rYJBI (0.4163), with all proteins classified as probable antigens (Figure 1c).

In addition, we conducted a detailed bioinformatic analysis of the protein sequences by comparing them with the corresponding sequences from other Rickettsia species. The results demonstrated that all proteins show substantial sequence similarity to proteins expressed by other members of the genus Rickettsia (Supplementary Figure S3).

### 3.2. Electrophoretic Profile of R. rickettsii Purified Proteins

**Figure 2:**
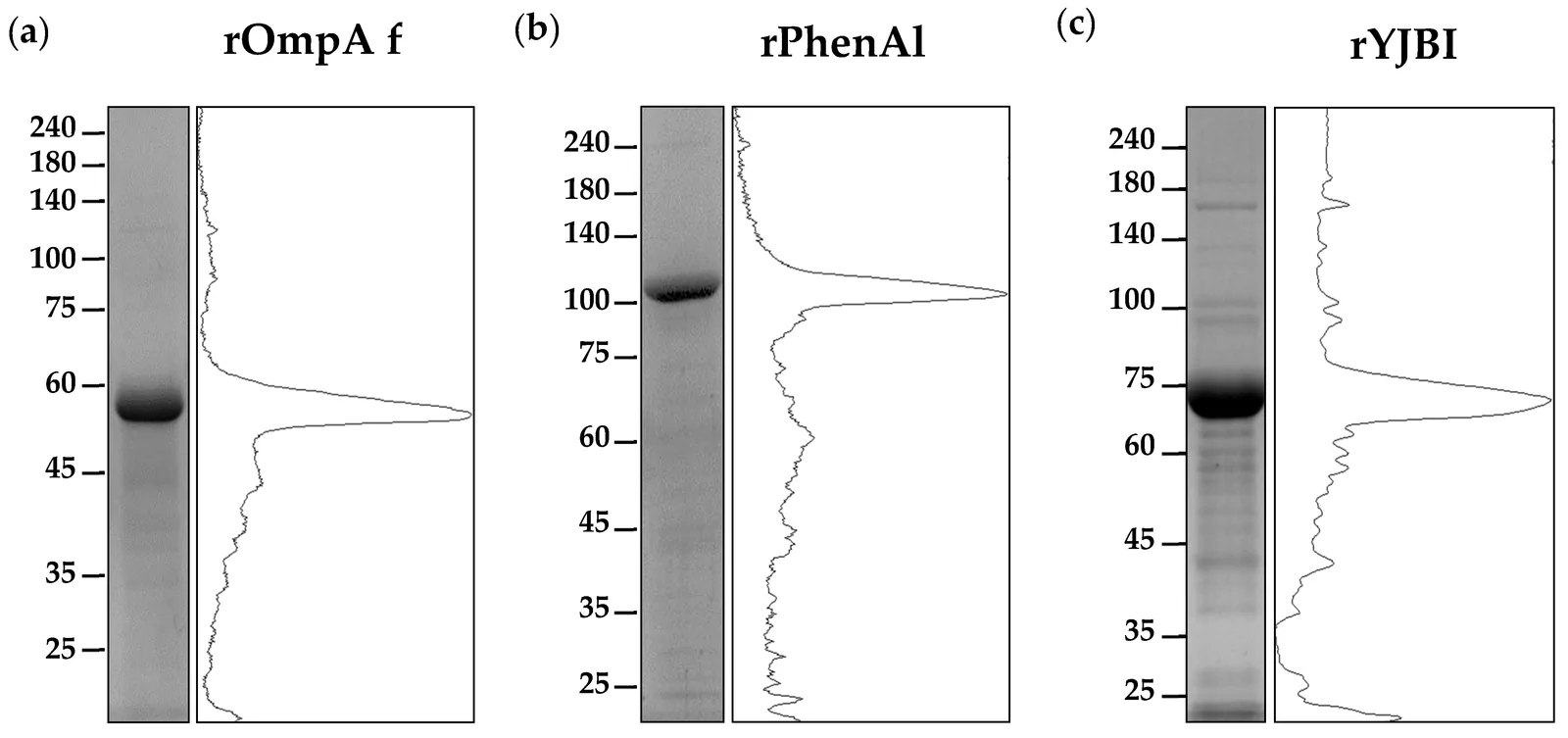
Production of the rOmpA f, rPhenAl and rYJBI proteins from *R. rickettsii*. (**a**–**c**) SDS-PAGE showing the isolated proteins (rOmpA f, rPhenAl and rYJBI) and densitometric profiles of the gels analyzed using ImageJ software. The TrueColor high-range protein marker is indicated on the left side of each SDS-PAGE. The imagens were recorded using iBright™ 1500 CL and adjusted for brightness and contrast.

Electrophoretic analysis of the isolated proteins revealed bands with molecular masses of 53 kDa for rOmpA f, 103 kDa for rPhenAl and 75 kDa for rYJBI (Figure 2). The differences observed between the computationally and experimentally calculated molecular masses are due to a C-terminal thioredoxin tag (Trx-tag) encoded by the pET-32a expression vector, which adds a 12 kDa peptide sequence to the total protein mass. Fractions isolated by FPLC using a HisTrap™ HP column demonstrated a relatively good degree of purity for the rOmpA f, rPhenAl and rYJBI proteins (Figure 2a–c).

### 3.3. Evaluation of the Humoral Immune Response After Immunization with the rOmpA f, rPhenAl and rYJBI Proteins in Mice

**Figure 3:**
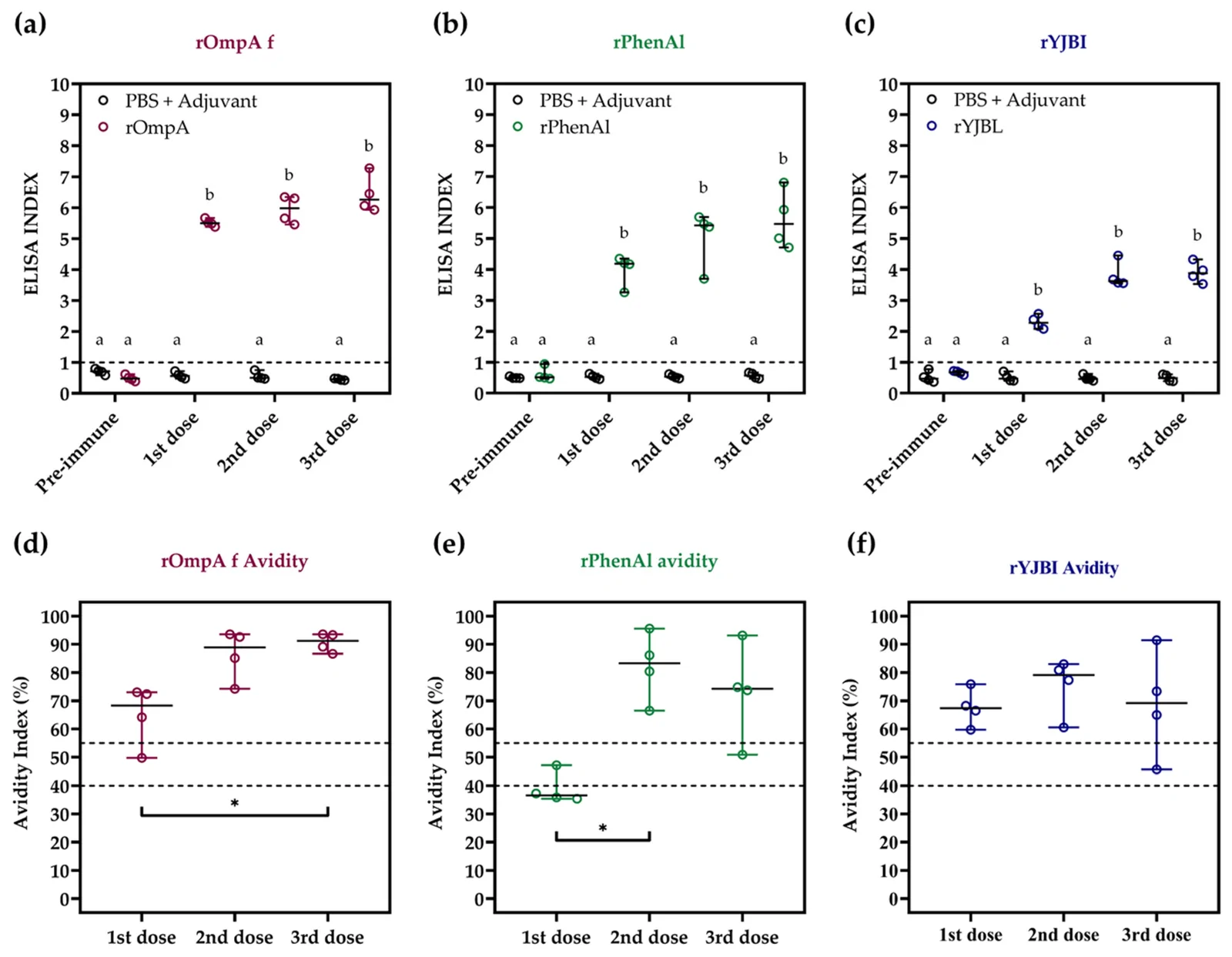
Evaluation of the humoral immune response of mice after immunization using the rOmpA f, rPhenAl and rYJBI proteins. (**a**–**c**) The ELISA index (EI) values are expressed as median with 95% confidence interval. The dashed lines represent the cut off value. Lowercase letters indicate statistically significant differences between immunized and mock groups. Groups sharing a letter do not show significant differences, while those with distinct letters do (*p* < 0.05), based on two-way ANOVA with the Geisser–Greenhouse correction and Tukey’s multiple-comparison test. (**d**–**f**) The avidity indexes (AI) were expressed as percentage and data reported as median with 95% confidence interval. The dashed lines represent predetermined arbitrary avidity levels defined as low (<40%), intermediate (40–55%) and high (>55%). Statistical significance between the AI was determined by paired one-way ANOVA with the Geisser–Greenhouse correction and Tukey’s multiple-comparison test or the Friedman test with Dunn’s multiple-comparison test when appropriate. * indicates *p* < 0.05.

The results showed that all recombinant proteins generated a strong humoral response after the third immunization dose, with reactivity reaching median EI values of 6.26 for rOmpA f, 5.47 for rPhenAl and 3.88 for rYJBI (Figure 3a–c). As expected, no reactivity was observed in the PBS-adjuvant group. Avidity analysis demonstrated that, throughout the immunization scheme, the recombinant proteins were able to induce IgG antibodies with increasing avidity (Figure 3d–f). The avidity index (AI) for rOmpA increased over time, with median values ranging from 68.27% to 91.23%. A significant increase in the AI for rOmpA was observed when avidity was evaluated between the first and third immunization doses (*p* = 0.0213). Similarly, an increase in avidity was observed for rPhenAl, with median values varying from 36.54% to 74.25%. A significant difference was observed only between the first and the second doses for rPhenAl (*p* = 0.04). No difference was observed between the first and the third doses. Although a small increase in AI mean was observed for YJBI between the first and the second doses, no significant difference in avidity was observed among the doses analyzed (Figure 3d–f).

### 3.4. IgG Antibodies from Swine Exposed to Rickettsia Species Recognize Recombinant Proteins

**Figure 4:**
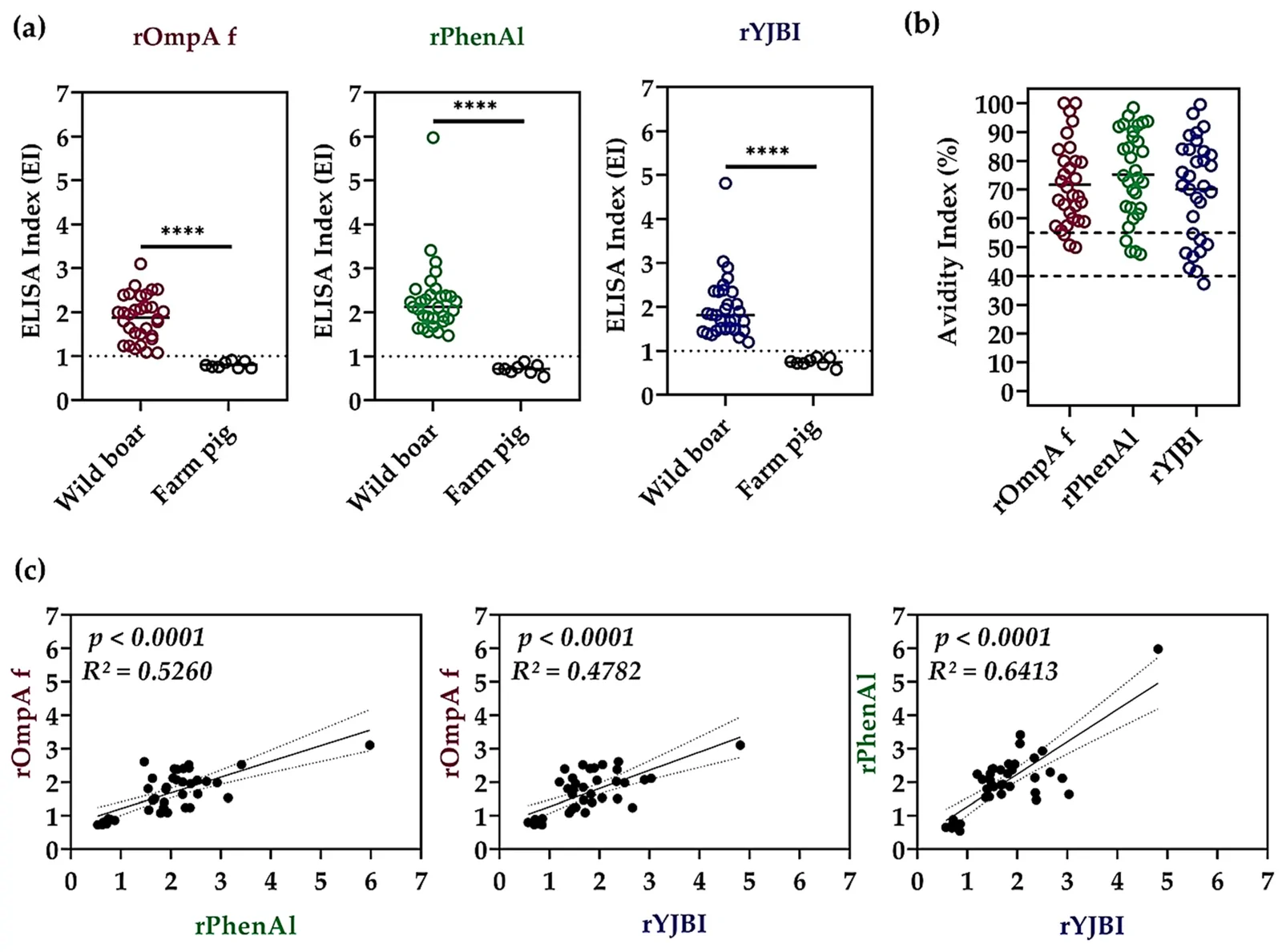
Reactivity of serum samples from farm pigs and wild boars against the rOmpA f, rPhenAl and rYJBI proteins. (**a**) Levels of IgG antibodies in serum samples from farm pigs (n = 8) and wild boars (n = 31) against the rOmpA f, rPhenAl and rYJBI proteins were evaluated by ELISA, and expressed as the ELISA Index (EI). Statistical significance was determined using the unpaired *t* test with Welch’s correction or the Mann–Whitney test, when appropriate. (**b**) The avidity indexes (AI) are expressed as percentages and the data are reported as the mean (n = 31). Statistical significance was determined by paired one-way ANOVA with the Geisser–Greenhouse correction and Tukey’s multiple-comparison test. (**c**) Analysis of correlations among the levels of seroreactivity against the rOmpA f, rPhenAl and rYJBI proteins using 31 serum samples from wild boars and eight samples from farm pigs. The calculated *p* and R^2^ values are indicated in the upper-left corner of each graph. **** indicates *p* < 0.0001.

A panel of 31 serum samples from wild boars that were reactive to spotted-fever-group rickettsiae (Supplementary Figure S2) and eight samples from farm pigs was used to assess seroreactivity (IgG antibodies) against the rOmpA f, rPhenAl and rYJBI proteins. As expected, when using serum samples from farm pigs, no reactivity against any of the recombinant proteins was observed, with median EI values of 0.77, 0.71 and 0.74 for rOmpA f, rPhenAl and rYJBI, respectively. In contrast, positive reactivity was observed in samples from wild boars, with median EI values of 1.95, 2.23 and 1.81 for rOmpA, rPhenAl and rYJBI, respectively (Figure 4a). A significant difference in seroreactivity between wild boars and farm pigs was observed for all three proteins analyzed (*p* < 0.0001). The positivity rate reached 100% for all three recombinant proteins, indicating that wild boars had been naturally exposed to *Rickettsia* spp. and produced specific IgG antibodies against the three recombinant proteins. In addition, avidity analysis revealed that serum samples from wild boars exhibited high-avidity IgG antibodies, with mean values of 71.64%, 75.22% and 70.11% for rOmpA f, rPhenAl and rYJBI, respectively (Figure 4b). In this analysis, no difference in avidity was observed among the proteins. When the recombinant proteins were analyzed in parallel, significant positive correlations were observed in swine IgG levels against rPhenAl and rOmpA (*p* < 0.0001), rYJBI and rOmpA (*p* < 0.0001), and rYJBI and rPhenAl (*p* < 0.0001) (Figure 4c).

## 4. Discussion

The diagnosis of rickettsiosis in the acute phase poses a great challenge since the disease is potentially fatal, with 70–85% of infections in Brazil caused by *R. rickettsii* [26]. Furthermore, the identification of domestic animals or natural wildlife reservoirs of *Rickettsia* spp. can be advantageous for defining the epidemiological scenario and adopting preventive strategies against this zoonosis. Real-time PCR has been applied to the diagnosis of RMSF; however, the gold standard for diagnosing rickettsiosis remains the indirect immunofluorescence assay (IFA) [5]. Therefore, investigations focused on new approaches and/or molecules from *R. rickettsii* may improve serological detection of RMSF or BSF during the early acute phase in humans and for identifying important natural wildlife reservoirs.

In this study, three *Rickettsia rickettsii* proteins (rOmpA f, rPhenAl, rYJBI) were immunochemically characterized. These candidates were previously identified by our group [12] using a phage-display approach, a powerful technology for obtaining molecular targets to be used in the diagnostic platforms and vaccines [27,28,29]. A dual-biopanning strategy using a 12-mer random peptide phage-display library (Ph.D.-12 NEB Library), followed by next-generation sequencing (NGS), was used to identify novel antigen candidates from *R. rickettsii*. In this approach, in vivo biopanning was performed in the *Amblyomma aureolatum* vector to enrich the phage populations that specifically bind to the tick target tissues (salivary glands, ovaries, midgut and Malpighian tubules), and the sequences identified by next-generation sequencing were analyzed bioinformatically against the complete *R. rickettsii* proteome, leading to the identification of seven immunoreactive regions/peptides from the *R. rickettsii* outer membrane protein A (OmpA). These immunoreactive peptides were selected and genetically engineered with flexible glycine-rich (GGGS) spacers to generate a small OmpA recombinant fragment (OmpA f). The same approach was also used to identify a new *R. rickettsii* hypothetical cell-surface protein, YJBI, as a promising candidate for binding to tick proteins (Supplementary Figure S1).

On the other hand, a phage-display approach based on humoral recognition was adopted to directly select immunoreactive candidates recognized by human antibodies. In this approach, purified IgG from pooled serum samples of patients with severe Brazilian Spotted Fever was used as the target for selecting reactive phages. Bioinformatic mapping of the most represented peptide motifs resulted in the identification of the phenylalanyl-tRNA synthetase (PhenAl) molecule as a potential new diagnostic target (Supplementary Figure S1).

Analyses performed to evaluate the molecular structures of these proteins demonstrated that rOmpA f and rYJBI have relatively compact three-dimensional structures with an abundance of small β-sheet secondary structures. The presence of β-sheets suggests that rOmpA and rYJBI are relatively stable molecules expressed by *R. rickettsii*, since at least one-quarter of all known proteins have molecular structures composed exclusively of β-strands and loops [30]. These findings were also supported by analysis using the ProtParam platform, which demonstrated low instability indices for rOmpA and rYJBI. The high molecular stability of *Rickettsia* spp. proteins may be an evolutionary adaptation, since these bacteria use different hosts (vertebrates and invertebrates) before infecting humans and causing RMSF or BSF.

In contrast, rPhenAl displays three globular domains maintained by a secondary arrangement of α-helices and β-sheets, which is consistent with the molecular features of other phenylalanyl–tRNA ligases [31,32]. In addition, the rOmpA f, rYJBI and rPhenAl proteins were also predicted to be potential antigens with long half-lives in *E. coli*, which led us to choose this organism as the expression system for these recombinant proteins.

Previous studies have demonstrated that OmpA is relatively similar among different *Rickettsia* spp. species and is an immunodominant protein that can be used as a potential candidate for the serological diagnosis of rickettsiosis [11,33]. In this context, we compared all three recombinant protein sequences from *R. rickettsii* with the corresponding sequences from other Rickettsia species. These results demonstrated high similarity to proteins expressed by other members of the genus Rickettsia. Therefore, the proteins used in our study are indeed derived from *R. rickettsii*, but we cannot state that these proteins are expressed exclusively by *R. rickettsii*, given that similar sequences are available in databases for other Rickettsia species.

Spotted-fever-group (SFG) rickettsiae express two immunodominant outer membrane proteins, OmpA and OmpB, which are conserved throughout the SFG and are thought to display a pivotal role in rickettsial pathogenesis [34]. OmpA plays an important role in bacterial adhesion to the host and is specific to SFG rickettsiae [34]. Under our experimental conditions, the immunization of mice induced high IgG production after injection, indicating that rOmpA f (fragment) is highly immunogenic. These data are in line with previous reports demonstrating that the whole rOmpA molecule induces antibodies reactive against *R. rickettsii* and provides protection against infection in a guinea pig model [35,36].

PhenAl (phenylalanyl–tRNA synthetase) is a cytoplasmic protein in which the organization of the α and β subunits is well conserved throughout evolution [37], although this molecule has not been studied in *R. rickettsii*. Interestingly, the rPhenAl protein from *R. rickettsii* was able to induce a strong humoral immune response, with high IgG titers detectable after the first dose, indicating that rPhenAl is a novel potential Rickettsia antigen for use in serological surveys.

Another novel molecule described in our work is the YJBI protein, an uncharacterized protein from *R. rickettsii*. YJBI was identified for the first time through a genomic approach and was subsequently detected in the predicted *R. rickettsii* proteome (personal communication; C.R.P); however, it had never been produced and/or purified. Therefore, this is the first study focusing on the immunochemical characterization of rYJBI from *R. rickettsii*. Immunization of mice with the rYJBI molecule elicited a strong antibody response, with high IgG titers after the first immunization, suggesting that it could also be a potential candidate for evaluation in diagnostic approaches and future vaccine development.

Accurate diagnosis of spotted fever (RMSF or BSF) is very difficult, especially at the beginning of the infection, given that the symptoms are nonspecific and similar to those of other diseases, including dengue, malaria, leptospirosis, viral hepatitis, meningitis and salmonellosis [3]. In 2023, Brazil experienced an outbreak of spotted fever on a farm in the state of São Paulo, with four deaths recorded, and at least one death was confirmed in 2024 [38]. Therefore, the evaluation of animal reservoirs of infection can be advantageous for defining geographic risk areas for BSF/RMSF and assist healthcare providers.

In this work, we evaluated IgG reactivity against the rOmpA, rPhenAl and rYJBI proteins using a well-characterized serum panel comprising samples from 31 wild boars with laboratory-confirmed seroreactivity by IFA and eight tick-naive farm pigs as negative controls. It is noteworthy that all wild boar serum samples, previously reactive via IFA to spotted-fever-group antigens (*R. rickettsii* or *R. parkeri*), showed 100% positivity in ELISA against the three recombinant antigens produced, suggesting that the recombinant molecules could be novel candidate antigens for detecting specific antibodies in wild animals naturally exposed to *Rickettsia* spp. Corroborating these data, high correlations were also observed between IgG reactivity against the PhenAl and YJBI proteins and reactivity against the OmpA molecule, a well-known protein expressed by SFG rickettsiae species that induces a potent antibody response [11,36].

A previous study showed that a peptide (mimotope) derived from the OmpA molecule had a strong ability to detect specific antibodies in opossum serum samples (85.7% sensitivity) by ELISA, although it had a weaker ability to discriminate between serum samples from horse and capybaras previously defined as IFA-positive and IFA-negative [9].

Since rickettsiosis is a worldwide problem with a growing incidence, knowledge about the immune response against novel *R. rickettsii* antigens, particularly regarding immunodominant molecules and new immunogens, may be valuable [39]. Current studies focusing on recombinant proteins for rickettsiosis are relatively scarce, and investigations focused on new *R. rickettsii* antigenic targets may be useful for improving human and veterinary serological diagnosis. The identification of novel antigenic targets and subsequent diagnostic validation studies may contribute to the development of improved diagnostic approaches for the accurate discrimination of infection caused by different Rickettsia species.

The novelty of this study was the demonstration of the reactivity and viability of these novel *R. rickettsii* antigens as a proof-of-concept for their use as candidate antigens in potential serological assays for detecting exposure to *Rickettsia* spp. in wild animal reservoirs. However, we recognize the limitations of the present study, including the lack of complete validation with a broad assessment of diagnostic sensitivity, specificity and other validation parameters, as previously evaluated by our group for COVID-19 [40], and the lack of a robust comparison using a large number of serum samples from wild animals from different regions or human serum samples.

Another limitation of our study, as reported for other *R. rickettsii* antigens, is the lack of discriminatory capacity at the species and clade levels among the characterized antigens. Notably, serum samples from wild boars that were exclusively reactive to either *R. rickettsii* or *R. parkeri* antigens by IFA also exhibited reactivity to the three antigens evaluated by ELISA, showing that these antigens cannot be used directly to discriminate between reactivity to different Rickettsia species.

Therefore, additional studies are necessary to validate new assays for diagnosing exposure to *Rickettsia* spp., including diagnostic assays for detecting neglected vector-borne pathogens in wild animal reservoirs.

## 5. Conclusions

In this study, novel recombinant proteins from *Rickettsia rickettsii* were expressed and characterized, demonstrating strong immunogenicity in mice and high seroreactivity in naturally exposed wild boars. These findings indicate that the evaluated antigens are suitable candidates for serological detection of Rickettsia exposure in animal hosts. Overall, the results expand the panel of potential serological markers for epidemiological studies of rickettsiosis.

## Data Availability

Data will be made available upon request.

## Supplementary Materials

The following supporting information can be downloaded at https://www.mdpi.com/article/10.3390/pathogens15090994/s1, Figure S1: Schematic overview of the workflow used to identify the *R. rickettsii* antigens characterized in this study. Two complementary approaches based on phage-display were employed to identify immunoreactive proteins from *Rickettsia rickettsii* [12]. (a) A biopanning approach conducted in vivo on *Amblyomma aureolatum*, coupled with next-generation sequencing (NGS) and bioinformatic analysis, led to the identification of the recombinant rOmpA f and rYJBI antigens. (b) The rPhenAl protein was identified through biopanning using IgG purified from patients with severe Brazilian Spotted Fever, followed by NGS and bioinformatics analysis. Figure S2: Analysis of serological reactivity in wild boar samples evaluated by indirect immunofluorescence assay (IFA) and indirect ELISA. Serum samples from 31 wild boars naturally exposed to tick infestation were evaluated by an in-house IFA using whole-cell *R. rickettsii* and *R. parkeri* antigens (spotted fever group) or by ELISA using the OmpA, PhenAl and YJBI proteins as antigens. Serum samples were considered positive by IFA or ELISA when they demonstrated antibody reactivity at a 1:64 dilution or an ELISA index > 1.0, respectively. Figure S3: Similarity analysis of the rOmpA f (immunodominant fragment), rYJBI and rPhenAl proteins with corresponding sequences from other Rickettsia species. The similarity among *Rickettsia rickettsii*, *R. parkeri*, *R. rhipicephali*, *R. amblyommatis*, *R. tillamookensis*, *R. bellii*, *R. typhi* and *R. prowazekii* sequences is presented as percentage identity (%). NI indicates not-identified/available sequences in the respective Rickettsia species.

## Abbreviations

The following abbreviations are used in this manuscript:

BSF	Brazilian Spotted Fever
RMSF	Rocky Mountain Spotted Fever
OmpA	Outer membrane protein A
PhenAl	Phenylalanyl tRNA ligase subunit beta
YJBI	Pentapeptide repeat-containing YjbI protein
LB	Luria–Bertani
PBS	Phosphate-buffered saline
