# Supplementary material for: Novel Rickettsia rickettsii Proteins with Potential Use for Serological Detection of Wild Natural Reservoirs
Source: Pathogens. 2026 Sep 20;15(9):994. doi: 10.3390/pathogens15090994 (PMC13610927; doi:10.3390/pathogens15090994)
Supplement: Supplementary file 1 [file pathogens-15-00994-s001.zip › pathogens-4431559-supplementary.pdf]

Supplementary Figure S1: Schematic overview of the workflow used to identify the *R. rickettsii* antigens characterized in this study. Two complementary approaches based on phage-display were employed to identify immunoreactive proteins from *Rickettsia rickettsii* [12]. (a) A biopanning approach conducted *in vivo* on *Amblyomma aureolatum*, coupled with next-generation sequencing (NGS) and bioinformatic analysis, led to the identification of the recombinant rOmpA f and rYJBI antigens. (b) The rPhenAl protein was identified through biopanning using IgG purified from patients with severe Brazilian Spotted Fever, followed by NGS and bioinformatics analysis.

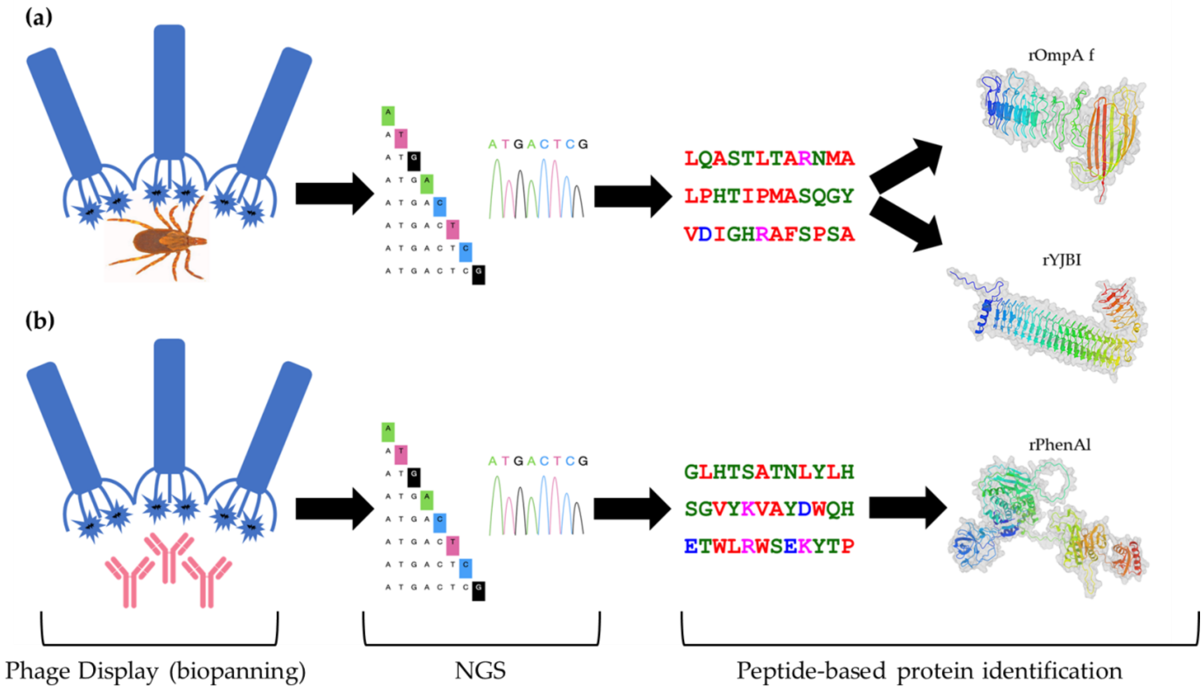

Supplementary Figure S2: Analysis of serological reactivity in wild boar samples evaluated by indirect immunofluorescence assay (IFA) and indirect ELISA. Serum samples from 31 wild boars naturally exposed to tick infestation were evaluated by an in-house IFA using whole-cell *R. rickettsii* and *R. parkeri* antigens (spotted fever group) or by ELISA using the OmpA, PhenAl and YJBI proteins as antigens. Serum samples were considered positive by IFA or ELISA when they demonstrated antibody reactivity at a 1:64 dilution or an ELISA index > 1.0, respectively.

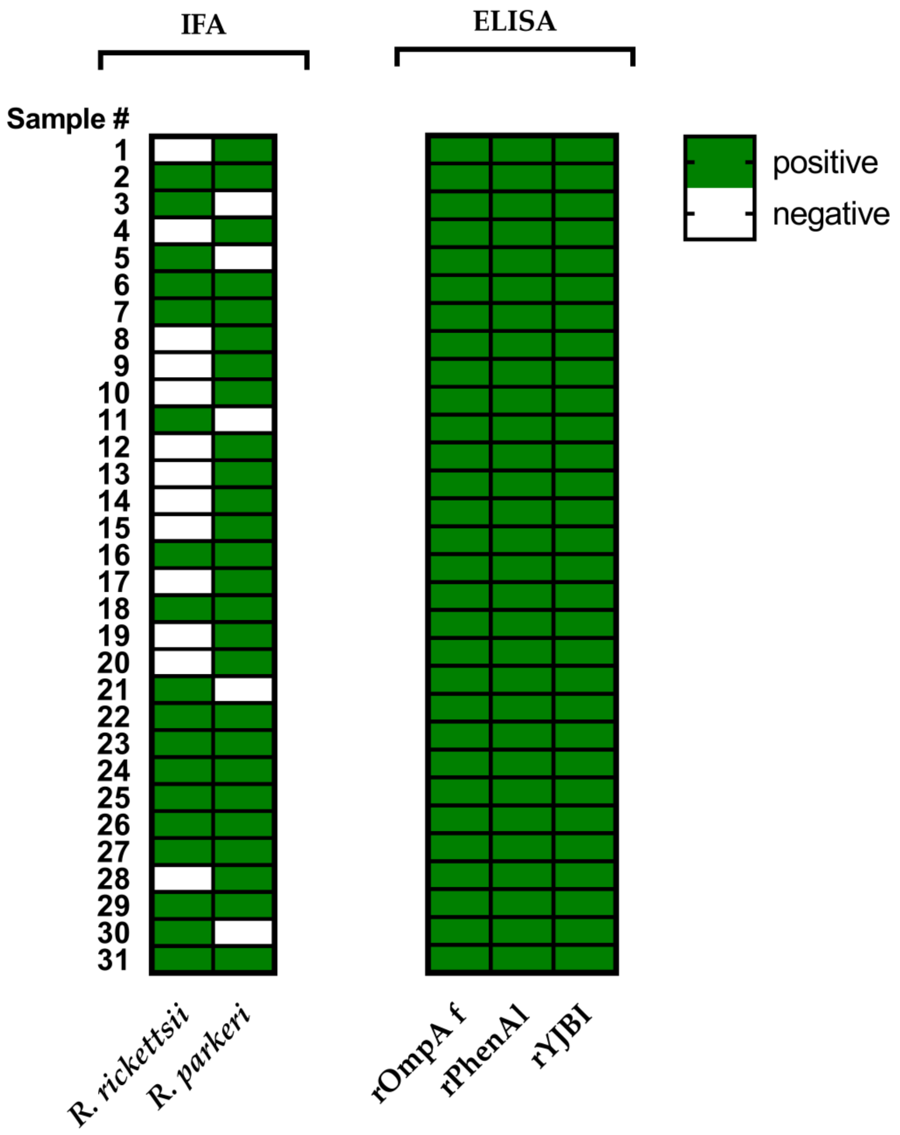

Supplementary Figure S3: Similarity analysis of the rOmpA f (immunodominant fragment), rYJBI and rPhenAl proteins with corresponding sequences from other Rickettsia species. The similarity among *Rickettsia rickettsii*, *R. parkeri*, *R. rhipicephali*, *R. amblyommatis*, *R. tillamookensis*, *R. bellii*, *R. typhi* and *R. prowazekii* sequences is presented as percentage identity (%). NI indicates not-identified/available sequences in the respective Rickettsia species.

	R. rickettsii	*R. parkeri*	*R. rhipicephali*	*R. amblyommatis*	*R. tillamookensis*	*R. bellii*	*R. typhi*	*R. prowazekii*
rOmpA f	71.65%	67.82%	66.28%	65.52%	67.92%	NI	NI	NI
rPhenAl	100%	98.53%	97.43%	96.09%	94.16%	71.25%	83.62%	84.47%
rYJBI	99.82%	98.07%	95.79%	96.65%	94.16%	73.86%	81.39%	81.46%

*NI: Non-Identified
